# The Assessment of Nutritional Status of Adolescents Aged 15-18 Years Using BMI Cutoffs and BMI Z Scores: A Secondary Analysis of National Family Health Survey-5 (2019-21) Data

**DOI:** 10.7759/cureus.59800

**Published:** 2024-05-07

**Authors:** Abdul Jaleel, Monica Chilumula, Surya Goud Chukkala Satya, Pooja Singnale, Usha Rani Telikicherla, Raghavendra Pandurangi

**Affiliations:** 1 Public Health Nutrition, Indian Council of Medical Research (ICMR) National Institute of Nutrition, Hyderabad, IND; 2 Maternal and Child Nutrition, Indian Council of Medical Research (ICMR) National Institute of Nutrition, Hyderabad, IND; 3 Nutrition Information Communication and Health Education (NICHE), Indian Council of Medical Research (ICMR) National Institute of Nutrition, Hyderabad, IND

**Keywords:** nutritional status of adolescents, thinness in adolescents, adolescent obesity, baz score, bmi for age, bmi, agreement, national family health survey, adolescent nutrition

## Abstract

Background: Appropriate assessment of nutritional status of adolescents as a population group is an important area of focus, considering the age of culminating growth, the size of the age group, the changing nutrition patterns, and as a window of opportunity for corrections before adulthood sets in. Nutritional status is now recognized to be a prime indicator of the health of individuals. The World Health Organization (WHO) emphasizes the importance of employing age- and sex-specific reference values (body mass index-for-age Z scores {BAZ} scores) for nutritional assessment in this age group. However, the National Family Health Survey (NFHS), the major source of data in India for the policymakers, reports the nutritional status of adolescents based on adult body mass index (BMI) cutoffs, which might not be as appropriate as compared to body mass index-for-age Z scores (BAZ) scores. Misclassification of nutrition status has impacts on public health policies, intervention programs, and long-term health outcomes for adolescents.

Methods: This secondary analysis of NFHS-5 data was performed with the objective of estimating the degree of agreement between BMI and BAZ cutoffs in classifying the nutritional status among Indian adolescents. The NFHS-5 data were collected from over 636,000 households across the country. Height and weight were measured for adolescents using standardized instruments. BMI and BAZ scores were derived to assess nutritional status. World Health Organization’s classifications were used to categorize nutritional status based on BMI and BAZ scores. The final analysis included data from 109,340 adolescents (13,040 males and 96,300 females) after excluding subjects having BAZ outliers and those whose age was 179 months or less.

Results: Substantial discrepancies emerged between the two methods. BMI classifications underestimated nutritional status in almost 30% of adolescents compared to BAZ. Over one-third of normal-weight individuals by BAZ are classified as thin by BMI. Conversely, nearly 78% of obese adolescents by BMI are classified as overweight by BAZ. The agreements between the classifications improved with age and were better among males.

Conclusion: This analysis highlights the limitations of BMI for assessing adolescent nutritional status and suggests that BAZ offers a more accurate and age-appropriate alternative.

## Introduction

The term "adolescence," originates from the Latin verb "adolescere," which encapsulates the notion of "growing to maturity." According to the World Health Organization (WHO), adolescents are persons in the age group of 10-19 years [1]. This age of transition, characterized by rapid physical and psychological development, witnesses the transformation from a dependent child to a self-sufficient adult [2]. According to recent statistics, approximately 1.2 billion adolescents worldwide constitute about 20% of the global population [3]. Among them, approximately five million adolescents reside in developing nations, and within India, adolescents represent around 16% of the overall population [4].

This period is commonly divided into early adolescence (10-14 years) and late adolescence (15-19 years). While early adolescence is dominated by pubertal changes, the later stages focus on sexual maturation and the assumption of adult roles and responsibilities [5]. Proper nutrition during adolescence is crucial for both immediate and long-term health outcomes, impacting not only the individual's current well-being but also their future health and the health of subsequent generations. Poor nutrition during this period can lead to delayed or stunted growth, which perpetuates the intergenerational cycle of undernutrition, i.e., undernourished females, in all likelihood, become undernourished mothers with a greater chance of giving birth to low birth-weight babies more prone to infections and growth failure. Additionally, inadequate dietary intake also increases the risk of developing non-communicable diseases (NCDs) later in life [6].

Nutritional status is now recognized to be a prime indicator of the health of individuals. The World Health Organization classified the nutritional status of adolescents using body mass index-for-age Z scores (BAZ scores). The classification is based on standard deviations from the median of a reference population. BAZ scores below -2 standard deviations indicate thinness or underweight [7]. BAZ scores between -2 and +1 standard deviations are considered within a normal or healthy weight range. BAZ scores between +1 and +2 standard deviations suggest overweight. BAZ scores equal to or above +2 standard deviations are indicative of obesity [8].

The National Family Health Surveys (NFHS) in India have traditionally relied on adult cutoffs for classifying nutritional status (undernourishment/overweight) in the 15-19 years age group [9]. This approach categorizes individuals with a BMI under 18.5 kg/m^2^ as thin, those between 25 kg/m^2^ and 29.9 kg/m^2^ as overweight, and those exceeding 30 kg/m^2^ as obese. However, this method faces limitations due to ignoring the crucial aspect of growth and development specific to adolescents.

According to the Comprehensive National Nutrition Survey conducted in India, (CNNS 2016-18) the prevalence of stunting, thinness, and overweight among adolescents was 26.4%, 24.1%, and 4.1%, respectively [10]. National Family Health Survey (NFHS-5) report reveals varying nutritional trends among 15-49-year-old males and females. Rural females exhibit a higher underweight prevalence (21.2%), while urban females show greater overweight or obesity rates (33.2%). In rural areas, males exhibit a higher prevalence of underweight (17.8%), while urban areas show elevated rates of overweight or obesity (29.8%) rates [11]. In the girls of age group 15-19 years, 39.7% reported to be thin (BMI<18.5), 4.2% to be overweight (BMI=25.0-29.9), and 1.2% to be obese (BMI=30.0 or above). For boys, the burdens were reported as 40.8%, 5.3%, and 1.2% respectively [12]. According to WHO, globally, 37 million children below five years of age and 390 million children and adolescents aged 5-19 years are either obese or overweight. It also reports the dramatic rise in overweight and obesity among children aged 5-19 years, increasing from just 8% in 1990 to 20% in 2022 [13].

The World Health Organization (WHO) emphasizes the importance of employing age- and sex-specific reference values for nutritional assessment in this age group. Recognizing this critical distinction, we re-analyzed the nutritional status of Indian adolescents using the WHO 2007 growth reference body mass index (BMI)-for-age and height-for-age growth charts. This approach offers a more refined picture of the prevalence of thinness, overweight, and stunting across various states, rural-urban divides, and wealth quintiles [8].

In India, with a significant adolescent population, understanding and addressing their nutritional status is paramount. The objective of this analysis was to estimate the degree of agreement in classifying the nutritional status among Indian adolescents aged 15-18 years (180-227 months) using BMI cutoffs as compared with BMI-for-age Z scores.

## Materials and methods

The NFHS over the years has emerged as a very important source of population data on health and nutrition, with its district-level data. The fifth round of NFHS was conducted in two phases, between June 2019 and April 2021 punctuated by the onset of the pandemic. It was conducted across 36 states and union territories and across 707 districts, covering 636,699 households. A detailed description of the survey methods is explained in the study report [12]. To summarize, a uniform sampling strategy was employed, giving weightage to the rural-to-urban divide, the percentage of population belonging to deprived communities, and literacy rates among females aged six years and above. Villages (rural) and Census Enumeration Blocks (urban) were drawn into the sampling frame of the primary sampling units (PSUs). Within each PSU, 22 households were selected with an equal probability of systematic selection after preparing the list of households. Data were collected through four survey questionnaires using Computer Assisted Personal Interviewing (CAPI) in local languages. Height and weight were measured for children aged 0-59 months, females aged 15-49 years, and males (in a subsample of households) aged 15-54 years. The Seca 874 digital scale was used to measure the weight of children and adults. The Seca 874 is a commonly used portable digital scale designed for field use with a weight capacity of up to 200 kg. It has a double display for easy reading and a tare function for weighing infants. The height of adults and children aged 24-59 months was measured with the Seca 213 stadiometer (Hamburg, Germany: Seca). The Seca 213 is a portable stadiometer, a tool for measuring height. It's lightweight (2.4 kg), folds up for easy transport, and doesn't require wall mounting for use. It reaches a height of up to 205 cm.

Inclusion and exclusion criteria

All the subjects willing to participate from the selected households were included in the survey. For this analysis, all subjects who were aged 15 to 18 years were included for analysis. Individuals who were not available at the time of survey and those who didn’t consent were excluded. For the analysis, children who were not between 15 years and 18 years, i.e., less than 180 months or greater than 224 months were excluded. Also, children with BAZ scores beyond +6 and less than -6 were excluded.

The present secondary data analysis was undertaken after the authorization from the Demographic and Health Surveys (DHS) Program (authorization letter 194852, dated December 29, 2023). The survey was approved by the Institutional Review Boards of the International Institute for Population Sciences (IIPS) and the informed consent form (ICF), and all participants filled out their informed consent forms (ICFs).

Individual level data of males and females were collected from the DHS program (Men’s Recode and Individual Recode for women data), which was then filtered for narrowing down to the age group of interest. All the subjects whose age was calculated in the dataset (variables ha1 and hb1) as to be 15, 16, 17, or 18 years, were selected to be included in the final analysis. The WHO AnthroPlus software (Geneva, Switzerland) was used to derive the BAZ scores. Stata v17 (College Station, TX: StataCorp LLC) was used for statistical analysis. In BMI classification, the adolescents were classified as thin if the BMI was less than 18.5 kg/m^2^, as normal if it ranged between 18.5 and 24.99 kg/m^2^, as overweight when ranging between 25 and 29.99 kg/m^2^, and as obese if BMI was 30 Kg/m^2^ or above [14]. By BAZ classification, the adolescents were classified as thin if the BAZ score was less than -2, as normal if the score was between -2 and +1, overweight if between +1 and +2, and obese if above +2 [7]. The cutoffs for each of the classifications at various ages and genders are summarized in Table 1.

**Table 1:** BMI levels at cutoffs to define various indicators of malnutrition at different ages and genders by BMI classification and BAZ classification. BMI cutoffs will vary with age and gender in BAZ classification [7]. All BMI values are in kg/m^2^. BAZ: body mass index-for-age Z scores

Indicator	Age in years	BMI classification	BAZ classification boys	BAZ classification girls
Thinness	15	<18.5	<16.0	<15.9
	16	<18.5	<16.5	<16.2
	17	<18.5	<16.9	<16.4
	18	<18.5	<17.3	<16.4
	19	<18.5	<17.6	<16.5
Normal	15	18.5-25.0	16.0-22.7	15.9-23.5
	16	18.5-25.0	16.5-23.5	16.2-24.1
	17	18.5-25.0	16.9-24.3	16.4-24.5
	18	18.5-25.0	17.3-24.9	16.4-24.8
	19	18.5-25.0	17.6-25.4	16.5-25.0
Overweight	15	>25.0	>22.7	>23.5
	16	>25.0	>23.5	>24.1
	17	>25.0	>24.3	>24.5
	18	>25.0	>24.9	>24.8
	19	>25.0	>25.4	>25.0
Obesity	15	>30.0	>27.0	>28.2
	16	>30.0	>27.9	>28.9
	17	>30.0	>28.6	>29.3
	18	>30.0	>29.2	>29.5
	19	>30.0	>29.7	>29.7

**Figure 1:**
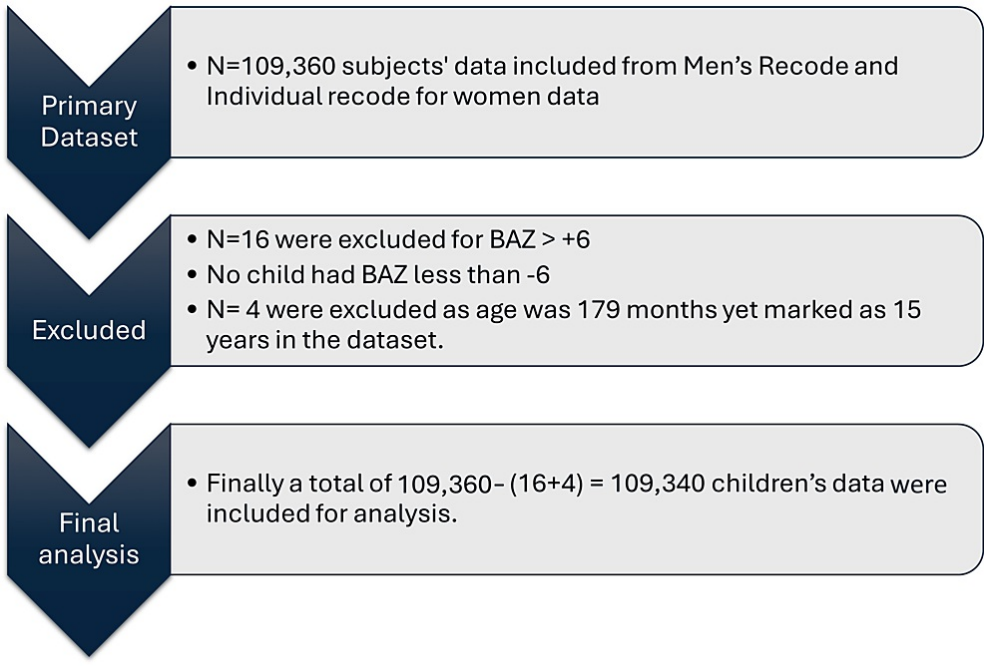
Flow diagram of children aged 15-18 years included in the analysis.

A total of 13,045 male and 96,315 female subjects had ages calculated in the data set to be between 15 and 18 years. Upon calculating their ages in months, four subjects (one male, three female) were found to be aged 179 months, which were excluded. Sixteen subjects (four male, 16 female) were excluded as the BAZ scores were over +6. As the dataset was already cleaned by NFHS, no exclusions were made for BMI outliers. Thus 13,040 males and 96,300 females, a total of 109,340 subjects were included in the final analysis (Figure 1).

Statistical analysis was done using Stata v16 and MS Excel. Distributions of age, gender, height, and weight of the sample were drawn. Proportions of thinness, overweight, and obesity as per BMI classification and BAZ classification were presented across the gender groups as percentages. The agreement was defined when the subject belonged to the same class in both classifications, e.g., subject was classified as obese in BMI as well as BAZ classification. If there was no agreement, it was assessed whether the BMI classification (used in DHS) was underestimating (if BAZ classified a subject as a higher class but BMI classified them as a lower class, e.g., obese by BAZ classification but normal/overweight by BMI classification) or overestimating (if BAZ classified a subject as a lower class but BMI classified them as a higher class, e.g., thinness by BAZ classification but normal/overweight by BMI classification). The agreements were presented as percentages and variability with age (in months) and gender was assessed as a trendline graph.

## Results

Table 2 shows the distribution of the sample based on age and gender. Nearly 12% of the sample was male and the rest was female. The representation was almost similar to the four groups of 12-month class intervals considered as belonging to the ages who had completed 15 years (180-191 months), 16 years (192-203 months), 17 years (204-215 months), and 18 years (216-227 months). Even within the genders, the distribution was similar. The mean age remained to be around 204 months (16 years and one month). Heights ranged between 87.3 cm and 206.5 cm and weights ranged between 15.0 kg and 142.8 kg. A wide range is expected for the age group with a usual growth spurt.

**Table 2:** Subject particulars - age and gender distribution.

Age	Males (n=13,040)	Females (n=96,300)	Total (n=109,340)
15 years (180-191 months)	3,198 (24.5)	24,017 (24.9)	27,215 (24.9)
16 years (192-203 months)	3,362 (25.8)	23,758 (24.7)	27,120 (24.8)
17 years (204-215 months)	3,116 (23.9)	22,753 (23.6)	25,869 (23.7)
18 years (216-227 months)	3,364 (25.8)	25,772 (26.8)	29,136 (26.7)
Mean age±SD (in months)	203.7±13.8	203.9±14.0	203.9±14.0
Range of height (in cm)	87.3-201.4	90.1-206.5	87.3-206.5
Range of weight (in kg)	17.7-134.4	15.0-142.8	15.0-142.8

The distribution of nutrition status classified using the two methods is shown in Table 3. Since the exercise was to compare the methods, the unweighted proportions were presented. It was observed that while nearly 57% of the adolescents were classified to be normal by BMI classification, more than 83% of them were considered to be normal as per the BAZ classification. While the proportions of adolescents classified as thin decreased by nearly 28% (absolute) upon using BAZ classification, the proportion of overweight increased. The obesity also is markedly overdiagnosed by BMI classification, nearly four times as much as by BAZ classification. The BAZ classification also differentiates the proportions of thinness, normalcy, and overweight more glaringly between males and females as compared to the BMI classification.

**Table 3:** Nutritional status of 15-18 year adolescents as per BMI and BAZ classifications across genders. The numbers and percentages are calculated to the column totals. Refer to Table 1 for cutoffs used for the classification. BAZ: body mass index-for-age Z scores

Variables	BMI classification			BAZ classification		
	Males	Females	Total	Males	Females	Total
Total sample	13,040	96,300	1,09,340	13,040	96,300	1,09,340
Thinness	5221 (40.04)	37,133 (38.56)	42,354 (38.74)	2,177 (16.69)	9,138 (9.54)	11,360 (10.39)
Normal	7,157 (54.88)	54,846 (56.95)	62,003 (56.71)	9,987 (76.59)	81,665 (84.8)	91,652 (83.82)
Overweight	550 (4.22)	3,452 (3.58)	4,002 (3.66)	845 (6.48)	5,264 (5.47)	6,109 (5.59)
Obesity	112 (0.86)	869 (0.9)	981 (0.9)	31 (0.24)	188 (0.2)	219 (0.2)

The agreement between both methods in classifying the nutrition status of adolescents is presented in Table 4. The table shows the number of subjects who were classified in each classification. For example, 11,360 subjects were classified as thin by both BMI as well as BAZ classifications, whereas in case of 30,994 subjects, the children were classified as normal by BAZ classification but as thin by BMI classification. The diagonal cells (from top left cell to bottom right cell) where both methods classify the subjects in same class are considered as agreements. The cells above the diagonal line are considered as underestimations by BMI classification and the cells under the diagonal cells depict overestimations by BMI classification. It is observed that both methods are in agreement with each other in the classification of nearly 70% of the adolescents whereas in most of the other cases, the BMI classification underestimates the nutritional status of one class less than the BAZ classification. A point of note is that there is 100% agreement of subjects classified as thin and obese by BAZ classification. The agreements are also high when BMI method classifies as normal (97.8%) and overweight (99.6%). The deviations are high in all the other diagnoses. More than one-third of the children classified as normal by the BAZ method are grouped as thin by BMI classification. Nearly 78% of the obese children as per BMI methods are overweight as per the BAZ method.

**Table 4:** Crosstabulation depicting number of subjects showing agreements of BMI and BAZ classifications on nutritional status of the 15-18 year adolescents. The agreement was defined when the subject belonged to the same class in both classifications, e.g., the subject was classified as obese in BMI as well as BAZ classification. *The diagonal cells (from top left cell to bottom right cell) where both methods classify the subjects in same class are considered as agreements. The cells above the diagonal line are considered as underestimations by BMI classification and the cells under the diagonal cells depict overestimations by BMI classification. BAZ: body mass index-for-age Z scores

Variables		BAZ classification				Total
		Thinness	Normal	Overweight	Obesity	
BMI classification	Thinness	11,360*	30,994	0	0	42,354
	Normal	0	60,641*	1,362	0	62,003
	Overweight	0	17	3,985*	0	4,002
	Obesity	0	0	762	219*	981
	Total	11,360	91,652	6,109	219	1,09,340

**Figure 2:**
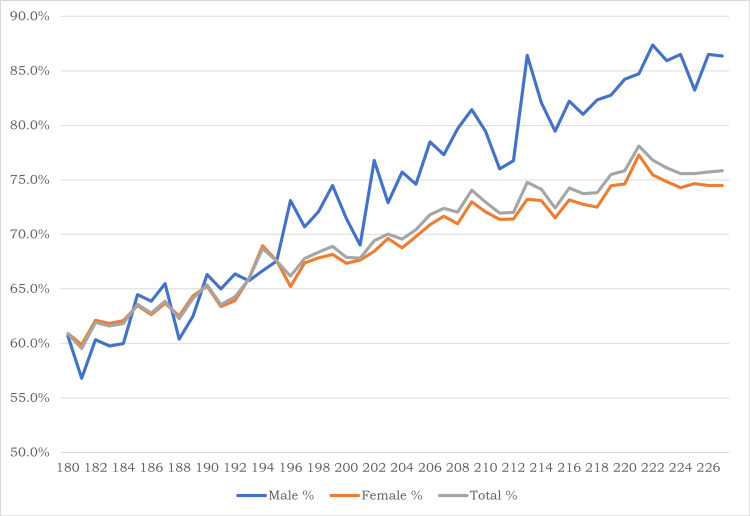
Trends of agreements between BMI and BAZ classifications on nutritional status of all the adolescents (n=109,340) with age among gender groups. The agreement was defined when the subject belonged to the same class in both classifications, e.g., a subject classified as obese in BMI as well as BAZ classification. BAZ: body mass index-for-age Z scores

The improvement in agreements with increasing age is illustrated in Figure 2. The percentage agreements (percentage of number of children with agreement among total number of children) were plotted for ages in monthly intervals for males, females, and combined. The agreements are seen to be better in males 74% than in females 69%. The agreements are less than two-thirds in the age group of 180-191 months while this nearly reaches up to three-fourths in the age group of 216-227 months (the table in appendix). The agreements were more or less similar and low in younger age groups, but the agreements tend to be higher among males than females and the difference in agreements between the genders also increases with age.

## Discussion

This study compared the efficacy of BMI cutoffs and BAZ scores in assessing nutritional status of adolescents aged 15-18 years. While BMI cutoffs are widely used, particularly in adults, BAZ scores offer a more nuanced approach by incorporating age- and sex-specific reference values. The findings revealed significant discrepancies in the prevalence of thinness, normal weight, overweight, and obesity between the two methods. This secondary analysis is an attempt to look at how the classification of adolescence by BMI method fares in comparison with the BAZ method. Since adolescence is the age of growth the WHO advocates for the use of BAZ scores as the method for assessing their nutrition status. The BMI cutoffs are more valid for adults who have already reached their near-maximum of high potential. The data set used was that of NFHS 5 which is standardized, meticulously collected, and has more than 1 lakh subjects in the age group.

The limitations of this analysis are that the number of females vastly outnumbers the males which is an inherent limitation from the data set that focuses more on the data collection of females. The data set also does not contain the details of adolescence between 10 and 14 years, hence that age group could not be included in the analysis. Additionally, the impact of socioeconomic background or other factors on the findings is not explored, to stick to the objectives of the analysis. Also in this analysis, unweighted proportions were presented, as the objective was not to present the burden of the disease but rather to compare the two methods.

The analysis encompassed 109,340 adolescents aged 15-18 years, revealing the following noteworthy findings: (1) discrepancies between BMI and BAZ classifications - a substantial discrepancy emerged between the two methods. BMI classifications underestimated nutritional status in almost 30% of adolescents compared to BAZ, resulting in notable overestimations of both undernutrition and obesity. (2) Underestimation of normal weight by BMI - a considerable proportion, nearly one-third of adolescents classified as "normal" by BAZ were identified as "thin" by BMI. (3) Overestimation of obesity by BMI - BMI significantly overestimated obesity compared to BAZ (0.9% vs. 0.2%), indicating potential misclassification of adolescents with higher muscle mass as obese. (4) Improved agreement with age - agreement between BMI and BAZ classifications increased with age, likely due to a more complete growth trajectory in older adolescents. (5) Gender differences: agreement between the two methods was higher for males (74.13%) compared to females (69.09%), possibly influenced by sex hormones and body composition disparities during adolescence.

Body mass index (BMI) is a widely used popular method for assessment of nutritional status. While BMI is uncomplicated in terms of calculation, it has its own limitations - primarily, it does not provide any information on body mass composition (composition and distributions of body fat, muscle tissue, and water content). So, the results are difficult to interpret in individuals with good musculature [15]. While in children, the interpretation of BMI is complicated due to the rapid growth and development of the body. In order to compare a BMI value with the norm, it is recommended that BMI standard deviation z score, age, and gender be considered [16].

The analysis clearly shows that the BMI method underclassifies the nutrition status of adolescence in nearly 30% of the subjects. Higher number of these children, mostly females, are classified as undernourished though by BAZ standards they might have been classified as to be within normal ranges. This is expected to grossly change the actual burden of undernutrition among adolescents in the country. On the other hand, the BMI classification also overestimates the diagnosis of obesity even though the global cutoffs are used, which are relatively higher levels than the Asian cutoffs. Using Asian cutoffs might have yielded an even higher burden of obesity and also a far higher difference with the BAZ method.

BMI might not be the most appropriate method for assessing nutritional status in adolescents, particularly for younger age groups. BMI's reliance solely on weight and height fails to account for body composition differences, leading to overestimation of thinness and obesity in adolescents who may have higher muscle mass. BAZ, on the other hand, incorporates both height and age, providing a more nuanced picture of nutritional status. This is particularly relevant for adolescents undergoing rapid growth and development, where weight may fluctuate without necessarily reflecting unhealthy changes. The higher agreement between BAZ and BMI for extreme categories (thin and obese) suggests that both methods might be suitable for identifying severely underweight or overweight adolescents. However, for normal weight, overweight, and at-risk categories, BAZ appears to offer a more accurate classification.

These findings align with prior studies. A secondary data analysis of nutritional assessment of adolescents (aged 15-19 years), available from the NFHS-3 and 4, reports that the adult cutoffs overestimate the thinness by almost 2.5-fold in the boys and four-fold in the girls [17]. Upon using the WHO 2007 growth reference as against the adult cutoffs, they reported that the thinness in boys and girls was 22.3% and 9.9% in NFHS-3 and 16.5% and 9% in NFHS-4, respectively, compared to 58.1%, 46.8%, 44.8%, and 41.9% reported by the NFHS. Overweight, which was reported as 1.7% in boys and 2.4% in girls by the NFHS-3, increased to 3% and 4.3%, respectively, on re-estimation using the WHO Growth reference. In NFHS-4, the same was 4.8% in boys and 4.2% in girls which increased to 6.2% and 5%, respectively, when estimated with WHO growth references. These revised estimates indicate a dramatically different adolescent nutrition status in India as against what is being reported. They also report these trends to be more consistent and plausible, such as in the older age groups. This analysis resonates with their thought that overestimation may not give an actual picture of the nutrition transition and the evolving double burden of malnutrition in the country. Another study that tried to compare different anthropometric indicators for assessing the nutritional status of adolescent girls in Delhi reports similar findings [18]. While BMI cutoffs categorize nearly 52% of these subjects as thin, this percentage drops to nearly 19% upon using BAZ scores. The proportion of overweight adolescent girls also increased from 8.3% to 10.6% and that of obesity increased from 1% to 3.9%.

The overdiagnosis of undernutrition can have its own implications. At a community level, these could lead to devising programs that may cause overfeeding which on one side would be expending valuable resources and on the other hand could have also increased the levels of overweight or obesity. At the individual level, the problem is more complex. Planning corrective measures to improve nutrition status in misclassified thin adolescents pushes the child towards obesity. Adolescence, being a formative age for one's feeding behaviors, may accentuate the problem of overeating. Recognizing this, the DHS Program (DHS-8) now reports adolescent-specific indicators of nutritional status with BAZ rather than BMI cutoffs for thinness, overweight, and obesity [19]. It is therefore prudent that BAZ scores are used instead of BMI cutoffs for the assessment of nutritional status of adolescents.

Similar analysis can be performed on datasets containing data on the whole spectrum of adolescence. Further analyses can be done to see the socioeconomic variabilities, inter-region, and inter-country variabilities. Also, prospective studies of these children can be done on yearly or decadal follow-ups to see how their nutrition status and susceptibility to NCDs can change.

## Conclusions

This analysis highlights significant discrepancies between BMI and BAZ scores in classifying adolescents' nutritional status. BMI underestimates nutritional status in nearly 30% of adolescents, particularly females, leading to potential overestimates of both undernutrition and obesity. Conversely, BAZ scores, which account for age and sex, provide a more nuanced picture. This is crucial during adolescence, a period of rapid growth and development. While both methods may be suitable for identifying overweight categories, BAZ offers a more accurate classification for normal weight, overweight, and at-risk groups. Transitioning to BAZ scores will provide a more accurate understanding of adolescent nutritional status, leading to better resource allocation and targeted interventions.

## Appendices

**Table 5:** Agreements of BMI and BAZ classifications on nutritional status of the adolescents across the age and gender groups. BAZ: body mass index-for-age Z scores

S. no.	Age group	Male	Female	Pooled
1	15 years	62.29	62.77	62.72
2	16 years	70.61	67.34	67.74
3	17 years	78.95	71.52	72.41
4	18 years	84.45	74.46	75.62
5	Pooled	74.13	69.09	69.7
